# Transpancreatic Precut Sphincterotomy for Biliary Access: The Relation of Sphincterotomy Size to Immediate Success Rate of Biliary Cannulation

**DOI:** 10.1155/2014/864082

**Published:** 2014-03-10

**Authors:** Lien-Fu Lin

**Affiliations:** Division of Gastroenterology, Department of Internal Medicine, Tungs' Taichung Metroharbor Hospital, Section 8, No. 699 Taiwan Boulevard, Wuqi District, Taichung 435, Taiwan

## Abstract

*Background*. Transpancreatic precut sphincterotomy (TPS) is an option for difficult common bile duct (CBD) access, and the reports are few, with immediate success rate varying from 60 to 96%. The description of relation between the size of TPS and the immediate success rate of CBD cannulation was not found in the literature. *The Aim of the Study*. To evaluate the relation of large TPS to immediate success rate of CBD cannulation. *Methods*. A retrospective analysis was performed in prospectively collected data of 20 patients. TPS was performed with traction papillotome in the main pancreatic duct (MPD) directing towards 11 o'clock. Needle knife (NK) was used to enlarge TPS in five patients, and the other 15 cases had large TPS from the beginning of sphincterotomy. Prophylactic pancreatic stent was inserted in 18 cases, with diclofenac given in 12 cases. *Results*. The immediate success rate of CBD cannulation was 90% and with an eventual success rate of 100%. The failure in one immediate CBD cannulation with large TPS was due to atypical location of CBD orifice, and the other failed immediate CBD cannulation was due to inadequate size of TPS. Complications included 3 cases of post-TPS bleeding and 3 cases of mild pancreatitis. *Conclusion*. TPS is an effective procedure in patients with difficult biliary access and can have high immediate success rate with large TPS.

## 1. Introduction

The initial success rate of selective CBD cannulation was reported to be 70% after a standard cannulation using guide wire and papillotome with the criteria of 5 attempts of cannulation [1]. Using double-guide wire (DGW) technique, the success rate varies from 47 to 73% with the pancreatitis rate of 14–38% [1–3]. Transpancreatic precut sphincterotomy (TPS) can be an alternative technique because the access is already in the MPD. The reports of TPS are few when compared to needle knife precut sphincterotomy, and detailed description of the size of TPS in relation to immediate successful CBD cannulation was not emphasized in the literature. The immediate success rate of TPS for biliary access varies from 60 to 96% [4, 5]. Diclofenac rectally administered and prophylactic pancreatic stent (PS) placement can reduce the incidence of postendoscopic retrograde cholangiopancreatography pancreatitis (PEP) [6]. We evaluate the primary outcome of immediate success rate of CBD cannulation with large TPS and secondary outcome of the complications with this new technique in our hospital.

## 2. Materials and Methods

**Figure 1:**
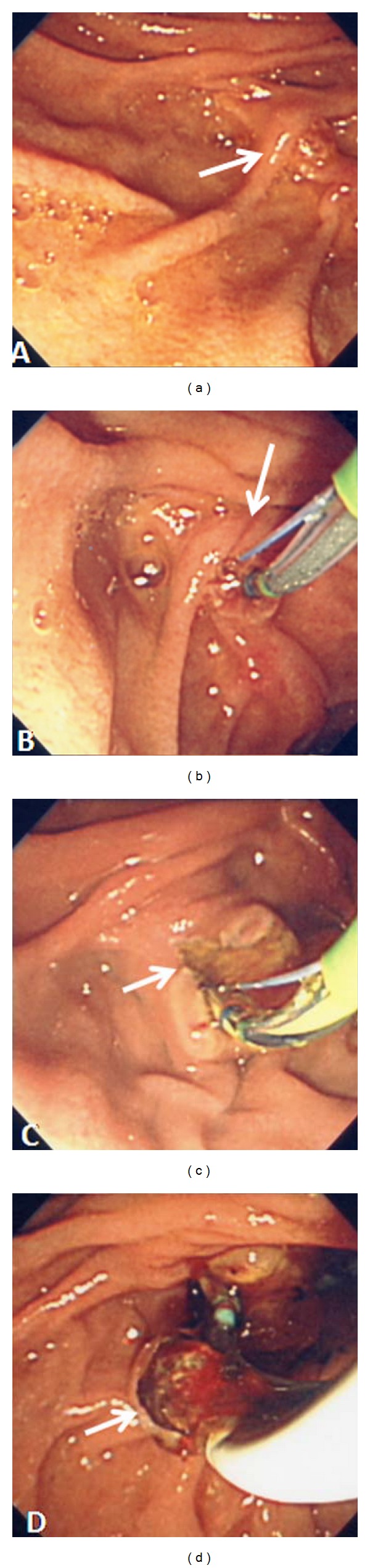
(a) Deviated papilla (arrow), (b) transpancreatic precut sphincterotomy in 11 o'clock direction (arrow), (c) CBD orifice (arrow) located left to the pancreatic duct occupied by sphincterotome, and (d) CBD stone extracted (arrow).

**Figure 2:**
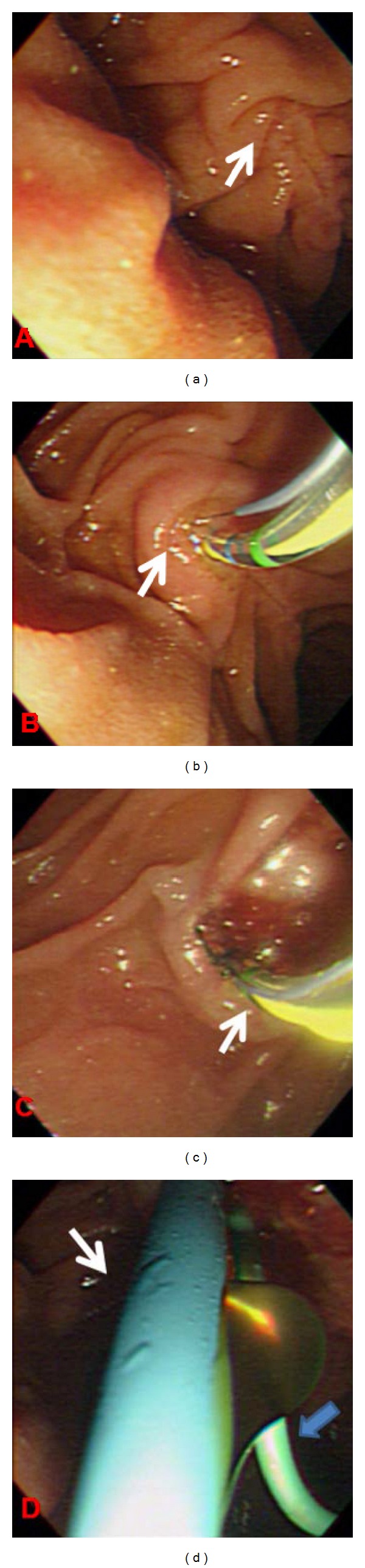
(a) Long papilla (arrow), (b) transpancreatic precut sphincterotomy in 11 o'clock direction (arrow), (c) CBD cannulation by sphincterotome (arrow), and (d) postbiliary and pancreatic stenting (white and blue arrows).

**Figure 3:**
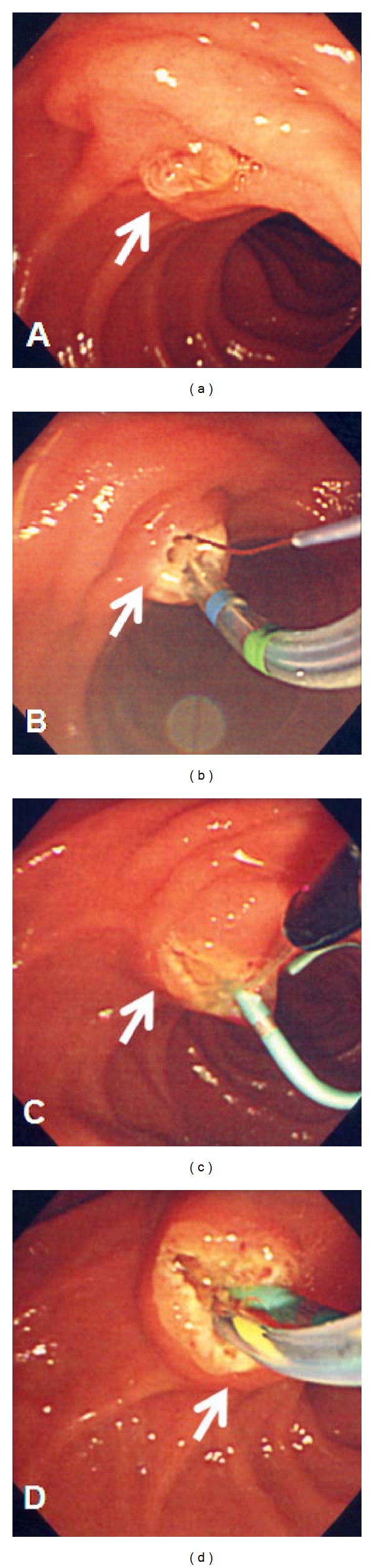
(a) Papilla (arrow), (b) transpancreatic precut sphincterotomy in 11 o'clock direction (arrow) with inadequate incision, (c) needle knife used to enlarge the incision (arrow), and (d) CBD cannulation (arrow).

**Figure 4:**
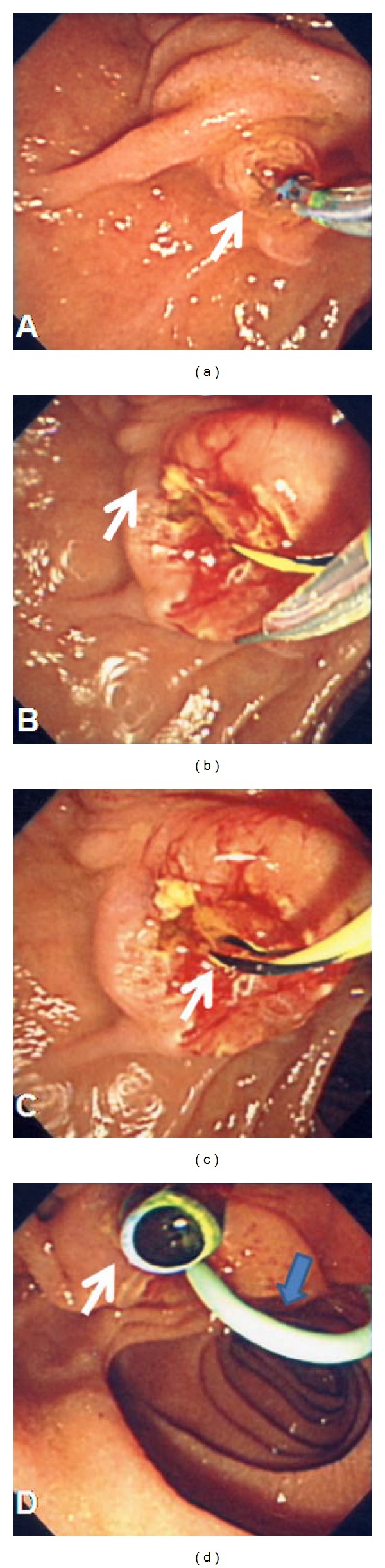
(a) Transpancreatic precut sphincterotomy in 11 o'clock direction (arrow), (b) large incision sphincterotomy (arrow), (c) successful CBD cannulation (arrow), and (d) postbiliary and pancreatic stenting (white and blue arrows).

Between January 2012 and October 2013, a retrospective analysis of prospectively collected data of 20 cases of TPS with inaccessible bile duct was conducted. There were 13 male and 7 female patients with a mean age of 54 ± 16 (25–82) years. The procedure was performed by a single operator who has experience on ERCP and endoscopic sphincterotomy (EST) for more than 1000 cases. A total of 112 cases of endoscopic retrograde cholangiopancreatography (ERCP) were performed during that period when TPS was started as a precut technique. The inclusion criteria were (1) failed selective deep CBD cannulation after 20 minutes and (2) successful access in the MPD. The exclusion criteria were (1) post-Billroth II gastrectomy cases and (2) failed access to the MPD. After access to the MPD, TPS was performed with the traction sphincterotome directing towards 11 o'clock, and CBD orifice is usually located left to the pancreatic duct after TPS (Figures 1 and 2). Needle knife was required to extend the TPS over the pancreatic stent (Figure 3) to get biliary access due to inadequate sphincterotomy in 5 cases. Large TPS incision up to superior margin of the papilla was performed in the other 15 cases (Figure 4). Pancreatic stent (PS) was placed either before or after the complete biliary procedure. The PS was removed after 4-5 days when there were no post-TPS bleeding and no spontaneous passage of the pancreatic stent. Diclofenac was given in patients who had balloon dilatation of the papilla or contrast injection into main pancreatic duct. Written consents were obtained from all of the patients. After TPS, serum amylase was tested on the next day and repeated on the 3rd day if the amylase level was elevated 3 times above the normal limit.

The instruments and current setting used were (1) duodenoscope (TJF 240, Olympus Optical Co., Ltd., Tokyo, Japan), (2) sphincterotome (Olympus KD-431Q-0720), (3) Boston Scientific Microvasive Jag wire, (4) Wilson Cook Zimmon pancreatic stent 5Fr-4 cm, (5) Wilson Cook Huibregtse HPC-3 needle knife, (6) electrosurgical generator (Olympus PSD 60), and (7) current setting (Endocut setting output of 120Watts and effect 2).

The definitions were as follows: (1) inaccessible bile duct was defined as failure after 20 minutes of CBD cannulation [7, 8], (2) complications of pancreatitis and bleeding were defined according to the criteria used by Cotton et al. [9], and (3) large incision sphincterotomy was defined as incision up to superior margin of the bulging papilla [4].

## 3. Results

**Figure 5:**
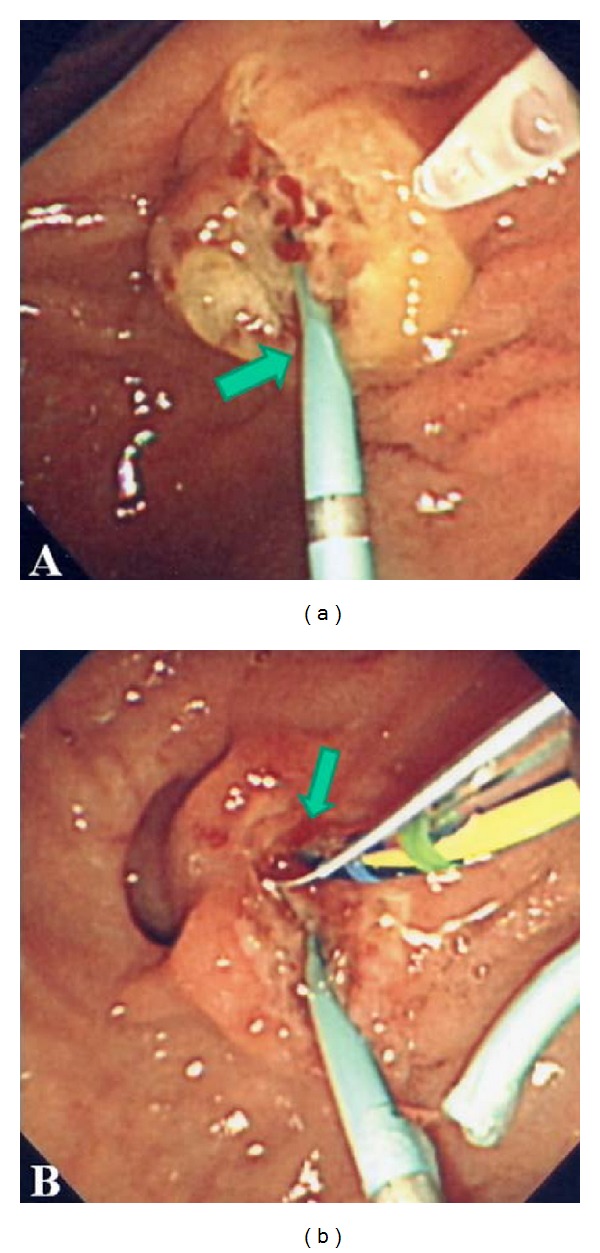
(a) Posttranspancreatic precut sphincterotomy with pancreatic stenting (arrow) and (b) CBD orifice located on the right side of pancreatic duct (arrow).

The results were shown in Table 1. The etiologies were 14 cases of common bile duct stone, 3 pancreatic head cancers, 1 bile leak, 1 biliary dilatation, and 1 chronic pancreatitis. The reasons for difficult selective CBD cannulation were periampullary diverticulum, long, and/or deviated papilla. Three cases had undergone trial of CBD cannulation with DGW technique but failed to get biliary access. Five cases needed additional needle knife sphincterotome to enlarge the TPS incision. All cases of large TPS incision had successful initial CBD cannulation except one owing to atypical location of CBD orifice (Figure 5). The immediate success rate of CBD cannulation after TPS was 90% and the eventual success rate was 100%. The other failed immediate CBD cannulation was due to inadequate size of TPS.

One massive delayed bleeding required surgery with uneventful recovery after failed endoscopic hemostasis and transarterial embolization. This severe bleeding case had undergone large TPS incision. One mild immediate bleeding was treated with endoscopic local epinephrine injection and clipping. The other mild repeated bleeding was managed endoscopically as well. Three cases of mild pancreatitis recovered within 2 days, though they had received prophylactic pancreatic stenting, and 2 cases had diclofenac given rectally. There was no perforation complication.

## 4. Discussion 

Techniques for difficult deep CBD cannulation include guide wire technique, DGW technique, and precut access. The success rate of CBD cannulation is not high in DGW technique ranging from 47 to 73% but the pancreatitis rate can be as high as 38% [1, 2]. Our unpublished success rate with DGW technique was 60% with a mild pancreatitis of 6%. Precut technique can have needle knife precut (free hand cutting at the papillary orifice or cutting the papilla over a pancreatic stent), needle knife fistulotomy above papillary orifice, and transpancreatic precut sphincterotomy (TPS) using traction papillotome [10].

TPS was first described by Goff et al. [11], and very few reports about comparison between TPS with needle knife sphincterotomy can be found [12]. TPS was reported to have a higher success rate and less complication compared with standard needle knife precut [13] and also a safe, effective procedure in patients with difficult bile duct access where classical sphincterotomy or needle knife procedures fail [14]. But on the other hand, Wang et al. conducted a multicenter prospective trial of TPS with needle knife precut sphincterotomy and concluded that there is no significant difference in success rate and complications [15].

The immediate success rate of TPS varies from 60 to 96% [4, 5]. No specific emphasis on or description about the size of TPS with the immediate success rate could be found in the literature. Although Goff reported a size of 5–7 mm of TPS achieving a success rate of 96%, but an initial success rate of only 60% was described by Akashi et al. with medium and large TPS [4]. Kapetanos et al. described an initial success rate of 75% without mentioning the exact length of TPS [16]. High initial success rate of 97% with large TPS was achieved by Halttunen et al. [17], and Kahaleh et al. also reported 85% success rate with large TPS [18]. Out of 20 cases of TPS in our hospital, 14 cases of large TPS incision at first attempt resulted in immediate successful CBD cannulation and faster procedure because no additional needle knife enlargement was needed. One large TPS that failed to get the biliary access was attributed to the atypical location of CBD orifice. Therefore, the key to a higher successful immediate biliary access is to incise a large TPS at the first attempt. The reason of requiring additional needle knife for extension of TPS in 5 cases was due to inadequate incision of TPS. Our immediate success rate of CBD cannulation was 90%, and eventual success rate was 100%.

The incidence of post-TPS bleeding varies from 3.7 to 5% [7, 16], our post-TPS bleeding was 15% which was a little higher and may be due to a small number of cases. Massive post-TPS bleeding was not reported in the literature. Severe postsphincterotomy bleeding was presumed to result from incision through an aberrant retroduodenal artery [19, 20], and the length of incision is not an important predictor of bleeding [21]. The cause of massive post-TPS (initial large TPS) bleeding in our series was attributed to have incised a larger branch of gastroduodenal artery after reviewing the angiography. More cases of TPS are needed for evaluation for post-TPS bleeding.

The incidence of pancreatitis without prophylactic stenting after TPS ranged from 5.5 to 21% [4, 7, 13] and reduced to 3.5% if prophylactic pancreatic stenting was performed [5]. We had 3 cases (15%) of mild pancreatitis, but all of them recovered in 2 days. All 3 cases had prophylactic pancreatic stenting done, and two had received diclofenac administration. The cause of pancreatitis in our series could be due to contrast injection into MPD or deep GW placement in the MPD. Severe post-TPS pancreatitis can occur without pancreatic stenting [3, 13]; therefore, pancreatic stenting is necessary in TPS procedure [6].

The advantages of large TPS are (1) definite complete unroofing of the papilla that makes CBD cannulation easier enabling a higher immediate success rate of biliary access and (2) probably low incidence of perforation which has been reported in two studies [5, 18]. The other credits of TPS are less technically demanding and easier to control the depth of cutting [13]. The limitations in this study are (1) small number of cases and (2) nonrandomized study for median or large TSP incision for comparison of immediate CBD cannulation success rate.

## 5. Conclusion

TPS is an effective procedure in patients with difficult biliary access and can have high immediate success rate with large sized TPS.

## Figures and Tables

**Table 1:** 

No.	Age	Sex	Diagnosis	Papilla	DGW	NKS	Success	Bleeding	P stent	Voren	Panreatitis
1	49	M	Pan cancer	Long	—	—	Yes	1 mild	Yes	—	—
2	47	F	CBDS	Long	—	—	Yes	No	Yes	100	—
3	44	M	Bile leak	Long	—	—	Yes	1 massive^#^	Yes	—	—
4	45	F	CBDS	Long, deviated	Yes	—	Yes	No	Yes	—	—
5	45	M	CBDS	Long, deviated	—	Yes	Yes	1 mild	Yes	—	Mild
6	32	F	CBDS	Deviated	Yes	Yes	Yes	No	Yes	—	—
7	77	M	CBDS	Long	—	Yes	Yes*	No	Yes	75	—
8	39	M	CCP	Long	—	—	Yes	No	Yes	100	—
9	25	M	CBDS	Deviated	—	Yes	Yes	No	Yes	100	—
10	72	M	CBDS	Long	Yes	—	Yes	No	Yes	75	—
11	56	F	CBDS	Long, deviated	—	—	Yes	No	Yes	—	—
12	56	F	CBDS	Long, deviated	—	—	Yes	No	Yes	100	Mild
13	58	M	CBDS	Deviated	—	—	Yes	No	Yes	75	—
14	56	M	Pan ccncer	Deviated	—	—	Yes	No	0	—	—
15	82	M	CBDS	PAD	—	Yes	Yes	No	Yes	50	—
16	70	F	BD (pap bx)	Long, deviated	—	—	Yes	No	Yes	75	Mild
17	73	M	Pan cancer	Deviated	—	—	Yes	No	0	—	—
18	70	M	CBDS	Long, deviated	—	—	Yes	No	Yes	100	—
19	64	M	CBDS	Deviated	—	—	Yes	No	Yes	100	—
20	29	F	CBDS	PAD	—	—	Yes*	No	Yes	100	—

DWG: double guide wire technique, NK: needle knife, P: pancreatic, Diclo: diclofenac, CBDS: common bile duct stone, BD: bile duct dilatation, Pap: papillary, PAD: periampullary diverticulum, *Second attempt of CBD cannulation, ^#^massive delayed TPS bleeding ended with surgery after failed endoscopic hemostasis and transarterial emboiization.

Long papilla: >2 cm and with difficult selective CBD cannulation.

Deviated papilla: difficult to adjust the papillary orifice in an en-face position.

Lower dose of diclofenac was given in older patients with mildly elevated renal function.
